# Intolerability to postoperative early oral nutrition in older patients (≥70 years) undergoing gastrectomy for gastric cancer: A case-control study

**DOI:** 10.1371/journal.pone.0251844

**Published:** 2021-05-19

**Authors:** Aelee Jang, Oh Jeong

**Affiliations:** 1 Department of Nursing, University of Ulsan, Ulsan, South Korea; 2 Department of Surgery, Chonnam National University Medical School, Gwangju, South Korea; Ohio State University Wexner Medical Center Department of Surgery, UNITED STATES

## Abstract

**Background:**

Postoperative early oral nutrition has increasingly been adopted for patients undergoing gastrectomy. However, intolerability to early oral nutrition remains a major concern, especially in older patients. This study aimed to investigate early oral nutrition intolerability in older patients who had undergone gastrectomy.

**Methods:**

We retrospectively reviewed 825 patients who had undergone gastrectomy for gastric carcinoma between 2017 and 2019. All patients received an oral diet on postoperative day 1. Patients were divided into older (≥70 years) and younger (<70 years) adult groups, and short-term outcomes and intolerability to oral nutrition were compared. Intolerability to early oral nutrition was defined as oral diet cessation due to adverse gastrointestinal symptoms.

**Results:**

Among the 825 patients (≥70 years, n = 286; <70 years, n = 539), 151 (18.3%) developed intolerability to early oral nutrition, of whom 100 patients were < 70 years old and 51 were ≥70 years old. The most common symptom causing intolerability was abdominal distension. The mean duration of fasting after developing intolerability was 2.8 ± 2.4 days. The incidence of intolerability in the older and younger adult groups was 17.8% and 18.6%, respectively (p = 0.799). In terms of sex, operative approach, gastric resection, lymph node dissection, reconstruction, and tumor stage subgroups, the older adult group did not exhibit a significant increase in intolerability. Postoperatively, the older adult group showed a higher incidence of systemic complications; however, anastomotic complications did not significantly differ between the two groups.

**Conclusions:**

Postoperative early oral nutrition can safely be adopted for older patients undergoing gastrectomy, with acceptable intolerability and surgical outcomes.

## Introduction

Despite a decrease in global incidence, gastric cancer is one of the most common malignant diseases and the leading cause of cancer-related mortality in East Asia [1]. Gastric cancer mostly affects the aged population. The proportion of patients aged ≥70 years has been reported to be approximately 25% in Korea [2]. Older patients may require careful management because of increasing operative risk due to lower reserves in physiologic functions, poor nutritional status, and frequent comorbid conditions [3–5].

Curative surgery is the mainstay of treatment for gastric cancer. With improving survival rates for gastric cancer, there is increasing interest in the quality of patient care. Enhanced recovery after surgery (ERAS) entails evidence-based multidisciplinary management that aims to reduce surgical stress and facilitate postoperative recovery [6]. ERAS has increasingly been adopted for patients with gastric cancer in recent years. Many studies have reported that ERAS can accelerate postoperative recovery and reduce the length of hospital stay without increasing patient risk after gastrectomy [7].

Postoperative nutrition is a major determinant that affects postoperative recovery after abdominal surgery [8]. The ERAS guidelines recommend starting postoperative oral nutrition on postoperative day (POD) 1 after gastric surgery [9]. Several studies have suggested the feasibility of early oral nutrition after gastrectomy through demonstrating faster bowel recovery, a shorter duration of hospital stay, and no increase in postoperative complications [10]. However, few studies have addressed early oral nutrition tolerability after gastrectomy. Moreover, with increasing numbers of older patients, intolerance to early oral nutrition in older surgical patients is a potentially concerning issue. Therefore, this study aimed to investigate the tolerability of early oral nutrition in older patients who received postoperative oral nutrition on POD 1 after gastrectomy for gastric carcinoma.

## Materials and methods

### Patients

**Fig 1:**
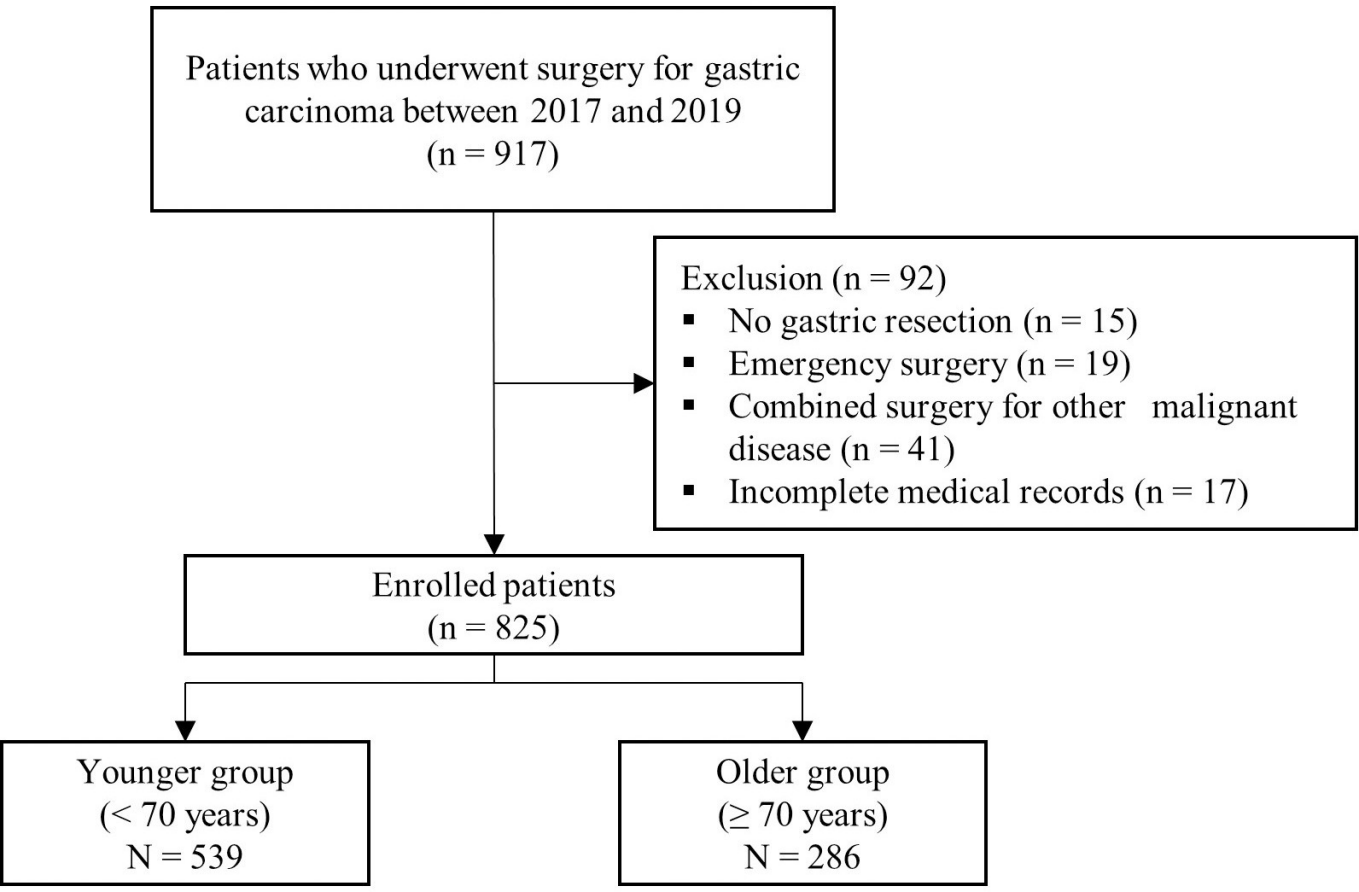
Flow diagram of patients.

This study was approved by the Institutional Review Board at our institution (Chonnam National University Hwasun Hospital), and the requirement for patient informed consent was waived. We retrospectively reviewed 917 consecutive patients who had undergone surgery for gastric cancer between 2017 and 2019 at a tertiary hospital (Chonnam National University Hwasun Hospital, South Korea). Eligibility criteria comprised patients who had received early postoperative oral nutrition after gastrectomy for gastric carcinoma. Of these, 92 patients were excluded, comprising those with no gastric resection (n = 15), those who had undergone emergency surgery due to bleeding or perforation (n = 19), those with combined surgery for other malignant diseases (n = 41), or those with incomplete medical records (n = 17). Finally, 825 patients were included in this study (median patient age, 63 years; inter-quartile range, 52–70 years). We divided the patients into older (≥70 years) and younger (<70 years) adult groups and compared the groups in terms of short-term surgical outcomes and intolerability to oral nutrition (Fig 1).

### Data collection and definitions

Patient clinical and pathological data were retrieved from a prospectively constructed gastric cancer database. This gastric cancer database includes all patients who receive surgery for gastric carcinoma. Patient demographic data, preoperative workups, operative results, patient outcomes including morbidity and mortality, and follow-up data are prospectively maintained using an electronic database that is accessible online. Clinicians manage this database, and the accuracy of imputed data is checked every month at a data review meeting. We collected data concerning patient demographic information (age, sex, body mass index, comorbidity, the American Society of Anesthesiologists [ASA] physiologic status, and medical history), operative results (curability, gastric resection, lymph node dissection, reconstruction, combined organ resection, operating time, and operative blood loss), pathologic results (tumor location, size, histologic differentiation, and tumor stage), and postoperative outcomes (diet start, passing gas, hospital stay, morbidity, and mortality).

The pathologic stage was based on the eighth edition of the American Joint Committee on Cancer tumor-node-metastasis (TNM) classification of gastric carcinoma [11]. Postoperative morbidity and mortality were defined as complications or deaths within 30 days postoperatively. Postoperative complications were classified as local or systemic, according to the site at which they developed. We used the Clavien–Dindo classification of surgical complications to classify complication severity [12].

The primary outcome of this study was intolerability to early oral nutrition. Intolerability was defined as an interruption of oral diet due to the development of adverse abdominal symptoms. At our institution, a diet record was completed for patients in which deviations from planned diet schedules and associated abdominal symptoms were noted. We collected data concerning the development of intolerability, the time of occurrence, related abdominal symptoms, and the duration of fasting after developing intolerability. For patients who developed intolerability, the day the oral diet resumed was recorded as the diet start day.

### Operative procedure and postoperative care

All the operations were performed by two surgeons specialized in gastric surgery. Gastric resection and lymph node dissection were performed based on gastric cancer treatment guidelines [13]. Laparoscopic surgery was indicated for clinically classified T1-2N0 tumors in the preoperative staging. After distal gastrectomy, Billroth I, Billroth II, or Roux-en-Y gastrojejunostomy was performed, as appropriate. After total gastrectomy, a simple Roux-en-Y esophagojejunostomy was performed.

The patients were postoperatively managed using ERAS, the details of which have been described in a previous study [14]. In our institution, the nutrition support team routinely assess a patient’s nutritional status prior to an operation, based on the clinical examination, diet history, weight loss, and laboratory data. We administer preoperative nutritional support using intravenous total parenteral nutrition or enteral nutrition for 4 to 7 days for patients diagnosed with severe malnutrition. Concerning postoperative nutrition, oral nutrition was administered from POD 1. We provided half a bowl of rice porridge with a side dish (approximately 120 to 150 cc, 300 kcal) three times a day. An additional oral nutritional supplement (200 kcal) was given to patients between meals. We instructed patients to gradually increase intake according to their tolerability.

### Statistical analyses

A Student’s t-test was used for continuous variables and chi-square or Fisher’s exact tests for categorical variables. All quantitative variables were considered as continuous data without grouping. Univariate and multivariate analyses of predicting factors for intolerability to early oral nutrition were performed using a binary logistic regression model. The risk of intolerability in older patients overall was examined as well as in subgroups in relation to various clinicopathological features. A Mantel-Haenszel test was used to examine the interaction between the subgroups. We also investigated the probability of intolerability according to age using the multivariable fractional polynomials (MFP) method to account for the nonlinear and asymmetric relationship between age and intolerability. All statistical analyses were performed using SPSS 22.0 version (IBM Corp, Armonk, NY, USA) and R 3.6.2, and two-sided p-values <0.05 were considered statistically significant.

## Results

### Patient characteristics

There were 286 and 539 patients in the older and younger adult groups, respectively. The clinicopathological characteristics of the two groups are presented in Table 1. The older group showed more frequent comorbidity (74.1% vs. 67.0%, p = 0.034), higher ASA scores (p = 0.006), and had a higher body mass index (24.4 vs. 23.7 kg/m^2^, p = 0.014). Preoperative anemia and hypoalbuminemia were more common in the older group, and open surgery (26.2% vs. 17.1%, p = 0.002) and D2 lymphadenectomies (43.7% vs. 33.4%, p = 0.004) were more frequently performed. Preoperative nutritional support was provided to 40 (7.4%) patients in the older group and 32 (11.2%) patients in the younger group (p = 0.068). In the final pathologic results, the older group showed more advanced TNM stages than the younger group.

**Table 1:** Clinicopathological characteristics.

	Younger group (n = 539)	Older group (n = 286)	p-value
Age (years)	56.8 ± 8.6	75.8 ± 4.4	
Sex			0.099
Male	359 (66.6)	174 (60.8)	
Female	180 (33.4)	112 (39.2)	
Body mass index (kg/m^2^)	23.7 ± 3.4	24.4 ± 3.2	0.014
ASA physiologic status			0.006
1	97 (18.0)	34 (11.9)	
2	367 (68.1)	192 (67.1)	
3	75 (13.9)	60 (21.0)	
Comorbidity	361 (67.0)	212 (74.1)	0.034
Previous abdominal operation	42 (7.8)	29 (10.1)	0.253
Preoperative anemia	79 (14.7)	95 (33.2)	<0.001
Preoperative hypoalbuminemia	7 (1.3)	10 (3.5)	0.034
Preoperative nutritional support	40 (7.4)	32 (11.2)	0.068
Operative approach			0.002
Open	92 (17.1)	75 (26.2)	
Laparoscopy	447 (82.9)	211 (73.8)	
Gastric resection			0.058
Distal gastrectomy	466 (86.5)	233 (81.5)	
Total gastrectomy	73 (13.5)	53 (18.5)	
Lymphadenectomy			0.004
D1+	359 (66.6)	161 (56.3)	
D2	180 (33.4)	125 (43.7)	
Combined organ resection	29 (5.4)	15 (5.2)	1.000
Differentiation (differentiated)	231 (42.9)	177 (61.9)	<0.001
TNM stage*			0.001
I	398 (73.8)	173 (60.5)	
II	58 (10.8)	49 (17.1)	
III	69 (12.8)	50 (17.5)	
IV	14 (2.6)	14 (4.9)	

Data are expressed as mean ± standard deviation or n (%)

ASA, American Society of Anesthesiologists physiologic status

*The eighth edition AJCC TNM classification of gastric carcinoma

### Intolerability to early oral nutrition

In total, 151 (18.3%) patients developed postoperative intolerability to oral nutrition (Table 2). Most patients (78% of patients showing intolerability) developed intolerability in the first 3 days postoperatively. Of 151 patients who developed intolerability, 75 patients showed abnormal findings on plain abdominal radiographs that suggested gastric stasis or ileus. The incidence of intolerability did not significantly differ between the older and younger adult groups (17.8% vs. 18.6%, p = 0.799, odds ratio [OR] 0.95, 95% confidence interval [CI] 0.66–1.38).

**Table 2:** Intolerability to early oral nutrition.

	All (n = 825)	Younger group (n = 539)	Older group (n = 286)	p-value
Intolerability	151 (18.3)	100 (18.6)	51 (17.8)	0.799
Occurrence time	2.7 ± 1.5	2.7 ± 1.6	2.7 ± 1.3	0.959
POD 1	24 (2.9)	18 (3.3)	6 (2.1)	
POD 2	71 (8.6)	46 (8.5)	25 (8.7)	
POD 3	23 (2.8)	15 (2.8)	8 (2.8)	
POD 4	13 (1.6)	6 (1.1)	7 (2.4)	
POD 5	9 (1.1)	6 (1.1)	3 (1.0)	
POD 6	8 (1.0)	6 (1.1)	2 (0.7)	
POD 7	3 (0.4)	3 (0.6)	0	
Duration of fasting (days)	2.8 ± 2.4	2.6 ± 2.0	3.2 ± 2.9	0.225
Abdominal symptoms				
Abdominal distension	102 (12.4)	70 (13.0)	32 (11.2)	0.455
Gastric fullness	21 (2.5)	14 (2.6)	7 (2.4)	0.897
Vomiting	15 (1.8)	6 (1.1)	9 (3.1)	0.037
Nausea	15 (1.8)	11 (2.0)	4 (1.4)	0.511
Postprandial pain	11 (1.3)	8 (1.5)	3 (1.0)	0.756
Reflux	6 (0.7)	3 (0.6)	3 (1.0)	0.423
Hiccups	2 (0.2)	1 (0.2)	1 (0.3)	1.000
Diarrhea	2 (0.2)	2 (0.4)	0	0.546

Data are expressed as mean ± standard deviation or n (%)

POD, postoperative day

The most common abdominal symptom in both groups was abdominal distension. When comparing each abdominal symptom between the two groups, the incidence of vomiting was more frequent in the older group (3.1% vs. 1.1%, p = 0.037). The mean duration of fasting after developing intolerability was 2.8 ± 2.4 days, which did not significantly differ between the two groups.

**Fig 2:**
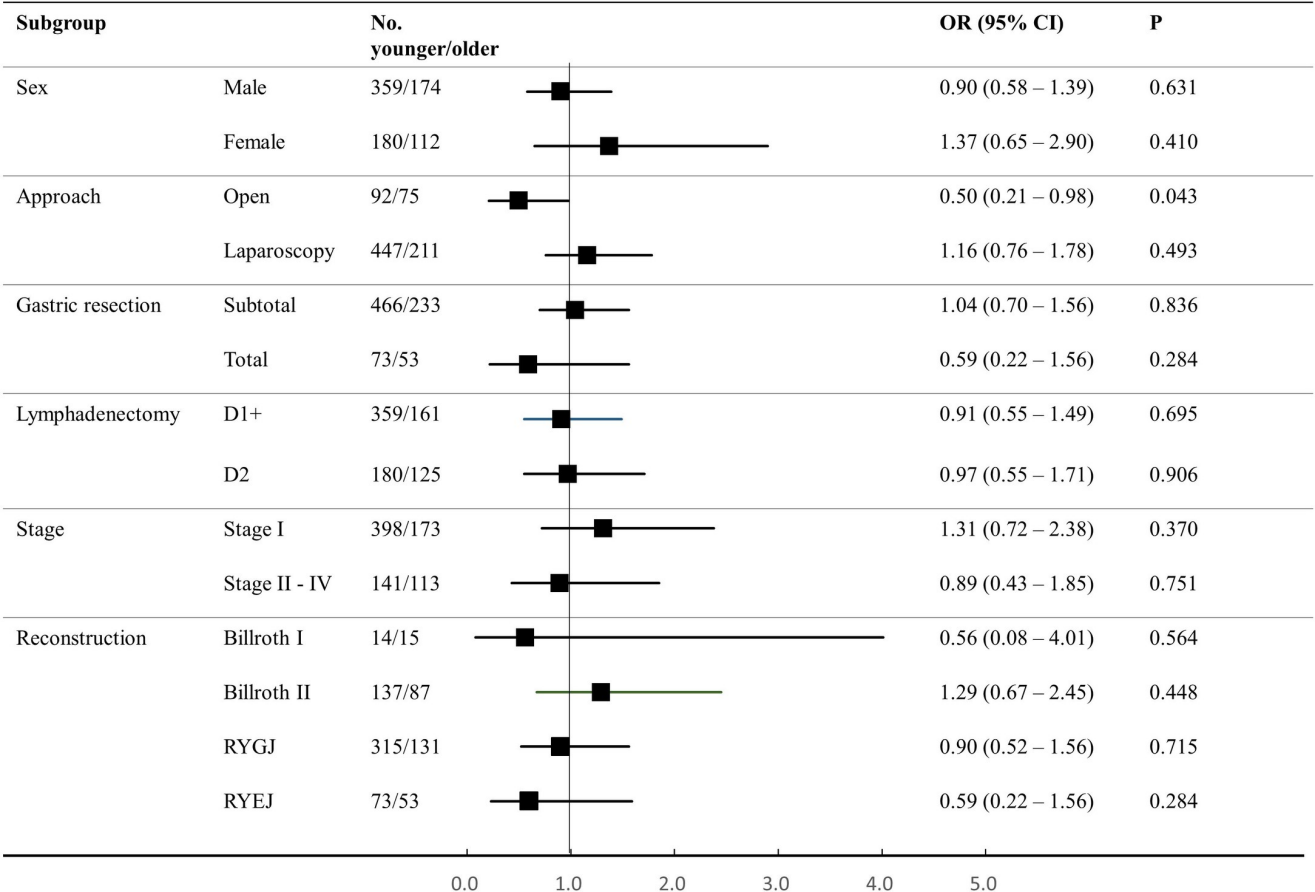
Intolerability in the older adult group in terms of different subgroups. The square box indicates the odds ratio for intolerability and the horizontal bar represents the 95% confidence interval. The older group showed no increase in intolerability in all subgroups. RYGJ, Roux-en-Y gastrojejunostomy; RYEJ, Roux-en-Y esophagojejunostomy.

Fig 2 shows odds ratios for intolerability in the older group according to the following subgroups: sex, operative approach, gastric resection, the extent of lymphadenectomy, reconstruction type, and tumor stage. The results show that the older group did not exhibit a significant increase in intolerability in any of these subgroups.

Table 3 shows univariate and multivariate analyses of factors that were associated with intolerability to early oral nutrition, in which preoperative (age, sex, comorbidity, previous upper abdominal surgery, tumor obstruction, and clinical stage) and operative factors (operative approach, the extent of gastric resection, operating time, and operative bleeding) were included. In the univariate analysis, male sex, tumor obstruction, and clinical tumor stage were associated with increased intolerability. Multivariate analysis revealed that only male sex (OR 3.56, 95% CI 1.98–6.40) was an independent predicting factor for intolerability to early oral nutrition.

**Table 3:** Univariate and multivariate analysis of factors affecting intolerability.

	Univariate			Multivariate		
	OR	95% CI	p-value	OR	95% CI	p-value
Age (years)	1.01	0.99–1.03	0.150			
Male sex	2.45	1.60–3.74	<0.001	3.56	1.98–6.40	<0.001
Comorbidity	0.86	0.59–1.26	0.449			
Previous upper abdominal surgery	0.80	0.41–1.60	0.523			
Tumor obstruction	3.04	1.39–6.62	0.005	2.44	0.83–7.15	0.105
Open surgery	1.49	0.98–2.24	0.060			
Total gastrectomy	0.94	0.57–1.54	0.790			
Operating time (h)	1.06	0.92–1.22	0.419			
Operative bleeding (dl)	1.03	0.99–1.07	0.168			
Clinical stage ≥II (vs. stage I)	1.83	1.17–2.88	0.008	1.67	0.96–2.91	0.070

CI, confidence interval; LND, lymph node dissection; OR, odds ratio

**Fig 3:**
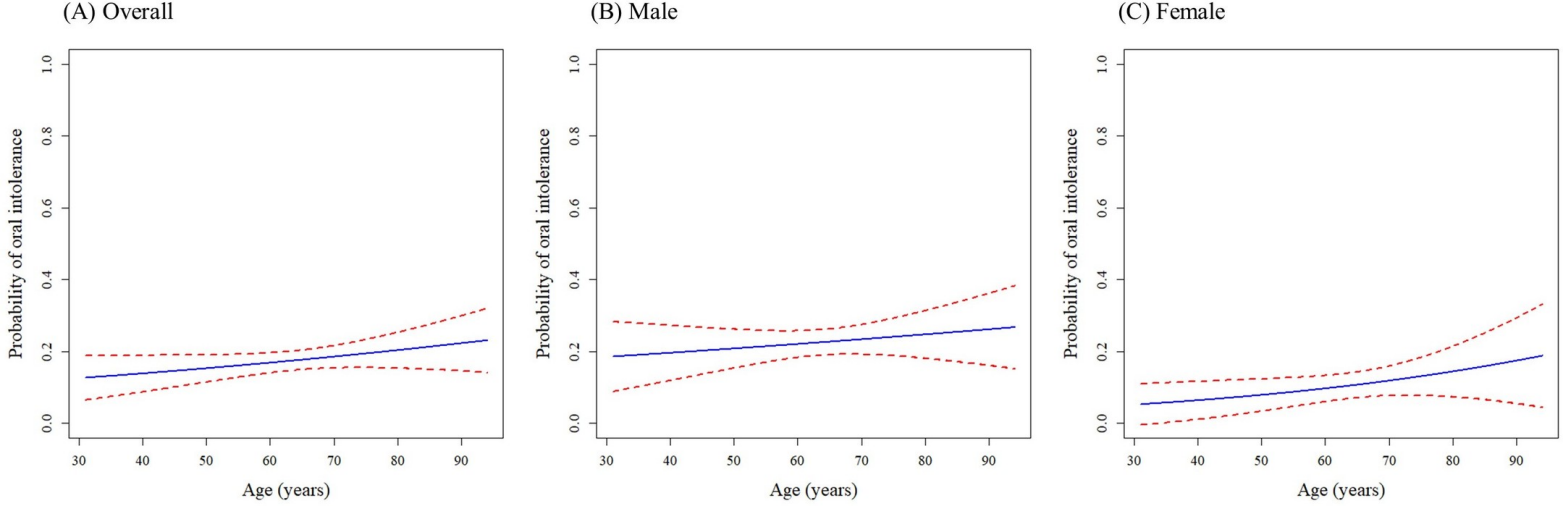
The multivariable fractional polynomials model showing the probability of intolerability to oral nutrition according to age. Probability was calculated using a regression model adjusting for sex. The dotted lines represent a 95% confidence interval of probability.

To account for the nonlinear and asymmetric relationship between age and intolerability, we applied the MFP method, maintaining age as a continuous variable. The regression model was estimated as follows:

$$logit(\pi_{i})=\beta_{0}+\beta_{1}age^{p_{1}}+\beta_{2}age^{p_{2}}+\beta_{3}Sex$$

where *π*_*i*_ was the probability of oral intolerance for individual *i*. *p*_1_ and *p*_2_ were the fractional powers for age. In the MFP model, age was not a factor significantly affecting oral intolerance (Fig 3).

### Short-term surgical outcomes

Table 4 shows postoperative outcomes in the older and younger adult groups. The older group showed a significantly higher incidence of systemic complications (13.3% vs. 5.9%, p < 0.001); however, local complications did not significantly differ between the two groups. The incidence of ≥grade 3 morbidity was significantly higher in the older group (4.5% vs. 1.7%, p < 0.001). The mean lengths of hospital stay in the older and younger adult groups were 9.5 and 8.3 days, respectively (p = 0.003). There was no significant difference in postoperative mortality. When we separately compared anastomosis-related complications, the incidence of anastomosis leakage, bleeding, and stricture did not significantly differ between the two groups.

**Table 4:** Postoperative outcomes.

	All (n = 825)	Younger group (n = 539)	Older group (n = 286)	p-value
Morbidity	168 (20.4)	94 (17.4)	74 (25.9)	0.004
Local complications	126 (15.3)	77 (14.3)	49 (17.1)	0.279
Systemic complications	70 (8.5)	32 (5.9)	38 (13.3)	<0.001
≥grade 3 morbidity*	22 (2.7)	9 (1.7)	13 (4.5)	0.015
Mortality	4 (0.5)	1 (0.2)	3 (1.0)	0.123
Hospital stay (POD)	8.7 ± 5.0	8.3 ± 4.5	9.5 ± 5.8	0.003
Anastomotic complications				
Leakage	5 (0.6)	4 (0.7)	1 (0.3)	0.664
Bleeding	4 (0.5)	2 (0.4)	2 (0.7)	0.613
Stricture	1 (0.1)	1 (0.2)	0	1.000

Data are expressed as mean ± standard deviation or n (%)

POD, postoperative day

*Clavien–Dindo classification of surgical complications

## Discussion

This study investigated intolerability to early oral nutrition among older patients (≥70 years) undergoing gastrectomy through comparing these patients with younger adult patients (<70 years). A strength of this study was that we prospectively collected information regarding intolerability using a diet record for all patients. We found that 18.3% of the 825 study patients were intolerant to early oral nutrition after gastrectomy, with no significant difference between the older and younger groups. Furthermore, anastomosis-related complications, such as an anastomotic leak, bleeding, or stricture, did not significantly increase in the older group. Our results suggest that postoperative early oral nutrition can be safely adopted for older patients after gastrectomy, with acceptable intolerability.

Many studies have demonstrated the safety and feasibility of early oral nutrition after gastrectomy [10]. However, previous studies have mostly focused on the length of hospital stay or postoperative complications to demonstrate the feasibility and safety of early oral nutrition. Many gastric surgeons remain concerned about tolerability to early oral nutrition because of relatively frequent abdominal symptoms after gastrectomy. Good tolerability needs to be guaranteed to apply early oral nutrition safely. We identified an acceptable level of intolerability during early oral nutrition after gastrectomy. Furthermore, most patients were able to resume an oral diet within 2 to 3 days after developing intolerability. These findings offer further support concerning the feasibility of early oral nutrition after gastrectomy.

Our univariate analysis found that male sex, tumor obstruction, and clinical tumor stage were associated with intolerability. Multivariate analysis of these factors indicated that only male sex was an independent predictor for developing early oral nutrition intolerability. The advanced tumor stage showed a trend toward increasing intolerability. Fronzo et al. [15] obtained similar results, reporting that male sex and operation type were factors predicting early oral nutrition failure after open colonic resection. The association between male sex and increased intolerability is possibly explained by differences in gastrointestinal physiology, dietary habits, and recovery perceptions between men and women [16]. Therefore, careful attention should be paid to male patients in relation to developing intolerability during early oral nutrition after gastrectomy.

Previous studies have reported that diet tolerability did not significantly differ between postoperative early oral nutrition and conventional feeding [17–19]. However, those studies used differing measurements to define intolerability, for example, nasogastric tube reinsertion, gastrointestinal-related morbidity, or the development of nausea or vomiting. Our study strictly defined intolerability in relation to oral diet, namely, interruption of at least one meal due to adverse gastrointestinal symptoms. This approach aimed to objectively identify deviation from an oral diet schedule related to gastrointestinal intolerability. Previous studies in colonic surgery have reported intolerability rates ranging from 25% to 27% in patients who received oral nutrition on POD 1 [20, 21]. Our findings concerning intolerability after gastrectomy were comparable to findings reported following other gastrointestinal surgeries.

Several studies have demonstrated the clinical benefits of early oral nutrition after abdominal surgery. In a meta-analysis by Lewis et al. [8] involving 13 randomized controlled trials (RCTs) concerning gastrointestinal surgery, early oral nutrition was found to have reduced hospital stay and decreased morbidity and mortality rates. A meta-analysis by Willcutts et al. [22] that included 8 RCTs and 7 non-RCTs concerning upper gastrointestinal surgery showed that early oral nutrition significantly reduced hospital stays without a significant increase in anastomosis leak, reoperation, or readmission rates. A review and meta-analysis of studies concerning early oral nutrition that focused on gastrectomy showed that early oral nutrition was associated with a shorter hospital stay and earlier bowel recovery without increasing postoperative complications [10]. However, most of these studies concerning gastrectomy involved limited numbers within a single institution. Currently, the ERAS guidelines recommend early oral nutrition after gastrectomy based on the fact that no trial has reported any adverse events [23]. To determine the clinical benefit of early oral nutrition in patients undergoing gastrectomy, further large multi-institutional RCTs are required.

Older surgical patients often manifest lower reserves in diverse physiologic functions, such as dysregulation of the immune and endocrine systems, decreased metabolism, poor nutrition status, or reduced physical activity. Moreover, older patients are more likely to exhibit various comorbid conditions, which may lead to increased postoperative morbidity [24]. Despite these concerns, our study findings demonstrated that early oral nutrition could be safely administered in older patients undergoing gastrectomy. We found that intolerability, as well as anastomosis-related complications, did not significantly increase in older patients. Subgroup analysis also showed that intolerability did not significantly increase in the older group in different operative and gastric resections. Therefore, we consider that being of older age in itself should not be a factor discouraging the implementation of early oral nutrition after gastrectomy.

In addition to intolerability, anastomosis safety is also a major concern that prevents surgeons from actively adopting early oral nutrition after gastrectomy. This concern is particularly relevant after total gastrectomy, as an esophagojejunostomy is considered to be more vulnerable to leakage. In this study, the incidence of anastomosis leakage with early oral nutrition was only 0.6%, which was comparable to that reported in previous studies of morbidity after gastrectomy [25]. In addition, we previously reported that postoperative morbidity, including anastomosis leakage, did not significantly increase in an early oral nutrition group after total gastrectomy [26]. An earlier animal study demonstrated that wound healing and strength in anastomosis could be increased with early feeding after upper gastrointestinal surgery [27]. Anastomosis safety associated with early oral nutrition has been well established in many studies, but may require further investigation in relation to total gastrectomy.

According to our protocol, oral nutrition is intended to provide a total of 1500 kcal per day. In this study, we could not provide relevant data to determine how adequate early oral nutrition was in terms of caloric delivery. However, we previously reported that most patients gradually increased their oral intake and could consume more than two-thirds of their meals after three to four PODs [28]. Therefore, we supplemented intravenous nutrition support for three PODs to endure proper nutritional support for patients.

This study had some limitations. First, this study was performed in a single, high-volume center with a well-organized multidisciplinary gastric cancer team, which may limit the generalizability of our results. Second, many patients were at an early disease stage and underwent laparoscopic surgery and limited lymph node dissection. Therefore, further validation may be required involving patients with advanced disease and undergoing open surgery. Overall, early oral nutrition should be implemented as a component of comprehensive ERAS and successful early oral nutrition requires a multidisciplinary team approach. Lastly, this study did not consider the statistical adjustment for multiple comparisons, which may be required for a more affirmative conclusion. Therefore, our findings indicate that the analysis and results are exploratory only.

## Conclusions

In conclusion, our study showed that intolerability to early oral nutrition after gastrectomy and other surgical outcomes did not significantly differ between older and younger adult patients. This finding suggests that early oral nutrition can be safely adopted for older surgical patients undergoing gastrectomy. Future research is recommended to determine the clinical benefits of early oral nutrition, focusing on older patients undergoing gastrectomy.

## Supporting information

S1 Dataset (SAV)
